# Differential Effects of Three Medium-Chain Fatty Acids on Mitochondrial Quality Control and Skeletal Muscle Maturation

**DOI:** 10.3390/antiox13070821

**Published:** 2024-07-09

**Authors:** Ryoichi Nishida, Shota Nukaga, Isao Kawahara, Yoshihiro Miyagawa, Kei Goto, Chie Nakashima, Yi Luo, Takamitsu Sasaki, Kiyomu Fujii, Hitoshi Ohmori, Ruiko Ogata, Shiori Mori, Rina Fujiwara-Tani, Hiroki Kuniyasu

**Affiliations:** Department of Molecular Pathology, Nara Medical University, 840 Shijo-cho, Kashihara 634-8521, Japan; g.m__r1@outlook.jp (R.N.); shota.nukaga@gmail.com (S.N.); isao_kawahara@a011.broada.jp (I.K.); y.miya1103@gmail.com (Y.M.); ilgfgtk@gmail.com (K.G.); c-nakashima@naramed-u.ac.jp (C.N.); lynantong@hotmail.com (Y.L.); takamitu@fc4.so-net.ne.jp (T.S.); toto1999-dreamtheater2006-sms@nifty.com (K.F.); brahmus73@hotmail.com (H.O.); pkuma.og824@gmail.com (R.O.); m.0310.s.h5@gmail.com (S.M.); rina_fuji@naramed-u.ac.jp (R.F.-T.)

**Keywords:** medium-chain fatty acids, mitochondrial quality control, mitophagy, reactive oxygen species, skeletal muscle

## Abstract

Nutritional interventions are one focus of sarcopenia treatment. As medium-chain fatty acids (MCFAs) are oxidized in the mitochondria and produce energy through oxidative phosphorylation (OXPHOS), they are key parts of nutritional interventions. We investigated the in vitro effects of three types of MCFA, caprylic acid (C8), capric acid (C10), and lauric acid (C12), in skeletal muscle cells. Compared with C10 and C12, C8 promoted mitophagy through the phosphatase and tensin homolog (PTEN)-induced kinase 1-Parkin pathway and increased the expression of peroxisome proliferator-activated receptor gamma coactivator 1-α and dynamin-related protein 1 to reduce mitochondrial oxidative stress and promote OXPHOS. Furthermore, the expression of myogenic differentiation 1 and myosin heavy chain increased in myotubes, thus promoting muscle differentiation and maturation. These results suggest that C8 improves mitochondrial quality and promotes skeletal muscle maturation; in contrast, C10 and C12 poorly promoted mitochondrial quality control and oxidative stress and suppressed energy production. Future animal experiments are required to establish the usefulness of C8 for nutritional interventions for sarcopenia.

## 1. Introduction

Sarcopenia is a pivotal challenge that develops with aging and deteriorates patients’ quality of life [1,2]. Moreover, various pathological conditions also cause myopenia. Among the pathological conditions, skeletal muscle loss reduces therapy tolerability and worsens disease prognosis in patients with cancer [3,4,5]. While increasing attention has been paid to treatments for sarcopenia by elucidating its mechanisms, these efforts remain insufficient.

Sarcopenia is caused by multiple factors, including reduced muscle cell regenerative capacity, an imbalance in protein turnover, changes in fat and fiber composition within muscles, increased reactive oxygen species (ROS) levels, mitochondrial dysfunction, and increased inflammation [6]. Our previous investigation on cancer-induced myopenia demonstrated the role of mitochondria in this condition and showed that increased ROS and suppressed oxidative phosphorylation (OXPHOS) promoted skeletal muscle catabolism [7]. We also previously reported that the combination of lauric acid (C12), a medium-chain fatty acid (MCFA), and glucose improved myopenia in a mouse model [7].

MCFAs are fatty acids with 6–12 carbon chains [8] in which mitochondrial β-oxidation does not depend on carnitine shuttles, unlike in long-chain fatty acids (LCFAs) [9]. Although reports have suggested that the degree of MCFA dependence on carnitine shuttles is low [10], in the living body, the oxidation of MCFAs that is not mediated by carnitine increases with exercise [11]. MCFAs have shown high utility in cancer cells and even in culture systems [12]. MCFAs can replace glucose as an energy source [13]. However, in mitochondria with poor quality control, such as cancer cells, they promote ROS production whereas, in normal mitochondria, OXPHOS can be used without producing ROS [12,14]. The cellular metabolism of MCFA is less dependent on fatty acid-binding proteins. Furthermore, it inhibits glycolysis, enhances lipogenesis and gluconeogenesis, does not exhibit protonophore activity, and does not impair electron transport in the respiratory chain [9].

However, C12 alone does not have a sufficient effect on cancer-related myopenia, and its odor makes it difficult for healthy people to consume [7]. Capric acid (C10) and caprylic acid (C8) have been reported to prevent frailty in older adults [15]. Moreover, mice fed a C8-enriched diet showed high locomotor activity and endurance [16]. However, little is known about the cellular-level effects of these MCFAs in cancer-related myopenia. Therefore, this study investigated the in vivo effects of C12, C10, and C8, particularly as mitochondrial substrates, in skeletal muscle cells.

## 2. Materials and Methods

### 2.1. Cells and Culture

The mouse myoblast cell line C2C12 (Riken Bank, Ibaraki, Japan) was cultured at 37 °C and 5% CO_2_ in Dulbecco’s modified Eagle’s medium (DMEM, 043-30085, FUJIFILM Wako, Osaka, Japan) containing 10% fetal bovine serum (173012, Sigma Life Science, St. Louis, MO, USA) and 2% penicillin (168-23191, FUJIFILM Wako, Osaka, Japan).

Before differentiation, the cells were treated for 48 h with C8 (50 or 200 μg/mL, FUJIFILM Wako), C10 (25 or 100 μg/mL, FUJIFILM Wako), or C12 (10 or 50 μg/mL, Tokyo Chemical Industry, Tokyo, Japan) dissolved in dimethyl sulfoxide (043-07216, FUJIFILM Wako, Osaka, Japan). MCFA treatment was only performed before differentiation, not during differentiation induction. To induce differentiation into myotube cells, the C2C12 cells were treated with DMEM containing 2% horse serum (S0900, Biowest, Riverside, MO, USA), 4.5 g/L glucose, and 1% penicillin for 48 h. Differentiation was morphologically confirmed by the presence of myotube formation. The regular and differentiation media did not contain any MCFAs.

### 2.2. Reverse Transcription–Polymerase Chain Reaction (RT-PCR)

To evaluate the mRNA expression level, RT-PCR was performed using total RNA extracted with TRI Reagent^®^ (TR118, Cosmo Bio Co., Ltd., Tokyo, Japan). Gene-specific primers (Table 1) were prepared by Sigma Genosys (Ishikari, Japan). The PCR products were detected via electrophoresis on 2% agarose gel and stained with ethidium bromide. β-actin was used as an internal control. Quantification of RT-PCR was performed using ImageJ software (version 1.52, Bethesda, Rockville, MD, USA).

### 2.3. Protein Analysis

After washing with phosphate-buffered saline (PBS), the C2C12 cells were suspended in M-PER mammalian protein extraction reagent (78501, Thermo Fisher Scientific, Tokyo, Japan) supplemented with protease and phosphatase inhibitors (1861280, Thermo Fisher Scientific) and placed on ice. Centrifugation was then performed for 20 min at 4 °C (15,000× *g*), and the supernatant was used as whole-cell lysate protein. The protein concentration was determined using a bovine serum albumin (BSA) protein assay kit (299-78421, FUJIFILM Wako, Osaka, Japan). Protein expression was analyzed via Western blotting. After adjusting the protein concentration, the proteins were separated via electrophoresis on polyacrylamide gel. After electrophoresis, the proteins were transferred to a polyvinylidene fluoride (PVDF) membrane. After being blocked with PBS containing 0.05% Tween 20 (Sigma) and 5% skim milk (Wako), the membrane was incubated with a primary antibody to label the proteins to be evaluated (Table 1). After reacting with the primary antibody, the membrane was reacted with a secondary antibody (Medical & Biological Laboratories, Tokyo, Japan) corresponding to each primary antibody, and the color was developed using a chemiluminescent detection reagent (Immobilon^®^ Forte Western HRP Substrate, Millipore, Darmstadt, Germany). The signals were observed using a Fusion Solo chemiluminescence detector. 7S. EDGE (M&S Instruments Inc., Osaka, Japan) and quantified using ImageJ software.

### 2.4. Mitochondrial Stress Test (Seahorse Assay)

C2C12 cells were cultured in filtered regular medium for 48 h. Oxygen consumption rates (OCR) of 1 × 10^3^ viable C2C12 cells per well were measured using a Seahorse XFe24 Extracellular Flux Analyzer with Seahorse XF24 FluxPaks (Agilent Technologies, Chicopee, ON, Canada). The Seahorse assays were carried out as follows: OCR in pmol/min was measured before (basal OCR) and after successive injections of 2 µM oligomycin (ATP synthase inhibitor), 0.5 µM carbonyl cyanide-p-trifluoromethoxy phenylhydrazone (CCP, an uncoupling protonophore), 0.5 µM rotenone (Complex I inhibitor), and 0.5 µM antimycin A (Complex III inhibitor). From the resulting data, we determined the OCR associated with respiratory ATP synthesis (oligomycin sensitive), the maximum OCR in FCCP-uncoupled mitochondria, the rotenone-sensitive OCR attributable to uncoupled Complex I activity, the antimycin-sensitive Complex II/III activity, and the OCR due to mitochondrial functions other than ATP synthesis, including OCR which is mitochondrial membrane potential driven (proton leak), non-respiratory oxygen consumption, and the respiratory “spare capacity” (excess capacity of the respiratory electron transport chain that is not being used in basal respiration).

### 2.5. Glycolytic Stress Test

The extracellular acidification rate (ECAR) of C2C12 cells was measured using a modified glycolytic stress test on a Seahorse XFe24 Extracellular Flux Analyzer with Seahorse XF24 FluxPaks. C2C12 cells (1 × 10^3^ cells/well) were plated in XF base medium (Agilent Technologies, Santa Clara, CA, USA) containing 200 mM L-glutamine and 5 mM 2-[4-(2-hydroxyethyl) piperazin-1-piperazine ethanesulfonic acid (HEPES), as recommended by the manufacturer for glycolytic assays. The sensor cartridge apparatus was rehydrated one day in advance by adding 1 mL XF Calibrant to each well and incubating it at 37 °C until needed. The injection ports of the sensor cartridge apparatus were loaded with 10 mM glucose, 2 µM oligomycin, 0.5 µM rotenone, 0.5 µM antimycin A (combined injection), and 50 mM 2-deoxyglucose, in chronological order of four injections, to produce the indicated final concentrations in the wells. Treatment with the rotenone/antimycin combination allowed for the assessment of the impact of electron transport on ECAR by respiratory acidification coupled with the passage of some glycolytic pyruvate through tricarboxylic acid (TCA) cycle to supply respiration.

### 2.6. Fluorescent Immunocytochemistry

C2C12 myotubes treated with each MCFA were fixed with 4% paraformaldehyde for 15 min at 4 °C, and the cell membranes were permeated with 0.1% TritonX-100/PBS. After blocking with 1% horse serum for 1 h, primary myosin heavy chain (MYH, 1:100) and myogenic differentiation 1 (MyoD, 1:100) were added and incubated overnight at room temperature. The cells were then washed three times with PBS and incubated with a secondary antibody conjugated to Alexa Fluor 594 (A21203; Thermo Fisher Scientific, Waltham, MA, USA) for 3 h at room temperature. The area of the MYH-positive myotubes [17] was measured using a fluorescence microscope (BZ-X710, Keyence, Osaka, Japan). Fused cell ratio (%) was defined as the MYH positive cell number divided by the total nuclear number stained by Hoechst33342 (Thermo Fisher) [17].

### 2.7. Mitochondrial Imaging

MitoROS (16052, Sigma, St. Louis, MO, USA) was used to evaluate ROS (mitochondrial hydroxy oxide), while mitoGreen (346-92061, Dojindo, Kumamoto, Japan) was used to assess mitochondrial volume. The luminescence intensity was quantified using a fluorescence microscope in luminance measurement mode (BZ-X710, Keyence).

### 2.8. Cell Growth

The effects of the three different MCFAs on the proliferation of skeletal myoblast C2C12 cells were examined. Cell growth was assessed using a 3-(4,5 dimethylthiazol-2-yl)-5-(3-carboxymethoxyphenyl)-2-(4-sulfophenyl)-2H-tetrazolium (MTS)-based Cell Titer 96 Aqueous One Solution Cell Proliferation Assay kit (Promega Corporation, Madison, WI, USA) as previously described. The absorbance of each well was measured at 490 nm using a multiscan FC microplate photometer (Thermo Fisher Scientific).

### 2.9. Mitophagy

To observe mitophagy, we used a Mitophagy Detection Kit^®^ (344-91901, Dojindo, Kumamoto, Japan). C2C12 cells were treated with 100 nM Mtphagy Dye for 15 min. After being washed with medium, the cells were then treated with 1 µM Lyso Dye and incubated for an additional 10 min. After being washed with the medium, the cells were observed under a fluorescence microscope (BZ-X710, Keyence).

### 2.10. Autophagy Flux Assay

To examine the effect of C8 autophagy on skeletal muscle differentiation, an autophagy flux assay was performed [18]. C2C12 myoblasts were pretreated with C8 (200 µg/mL) and chloroquine (CQ, 10 µM, HY-17589, Med Chem Express, Princeton, NJ, USA) for 48 h and then treated using the differentiation procedure described above.

### 2.11. Statistical Analysis

The obtained data were analyzed using one-way analysis of variance (ANOVA), and multiple comparisons were performed using Bonferroni correction. EZR version 1.60 was used for data analysis. The significance level for all statistical analyses was set at *p* < 0.05.

## 3. Results

### 3.1. Effects of the Three MCFAs on Skeletal Muscle Differentiation

**Figure 1:**
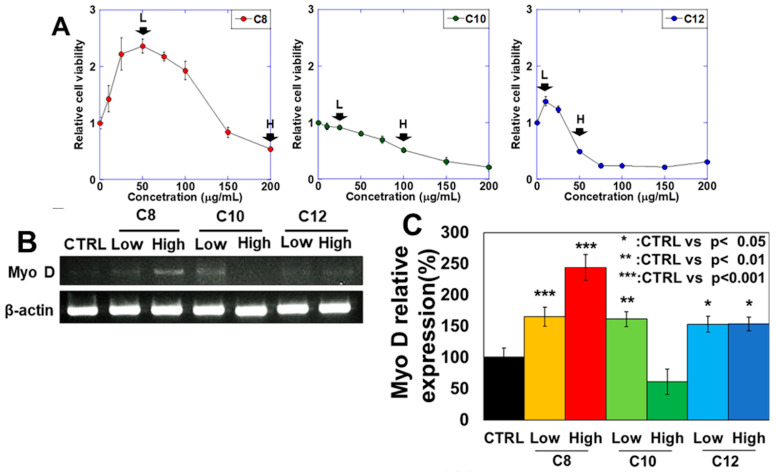

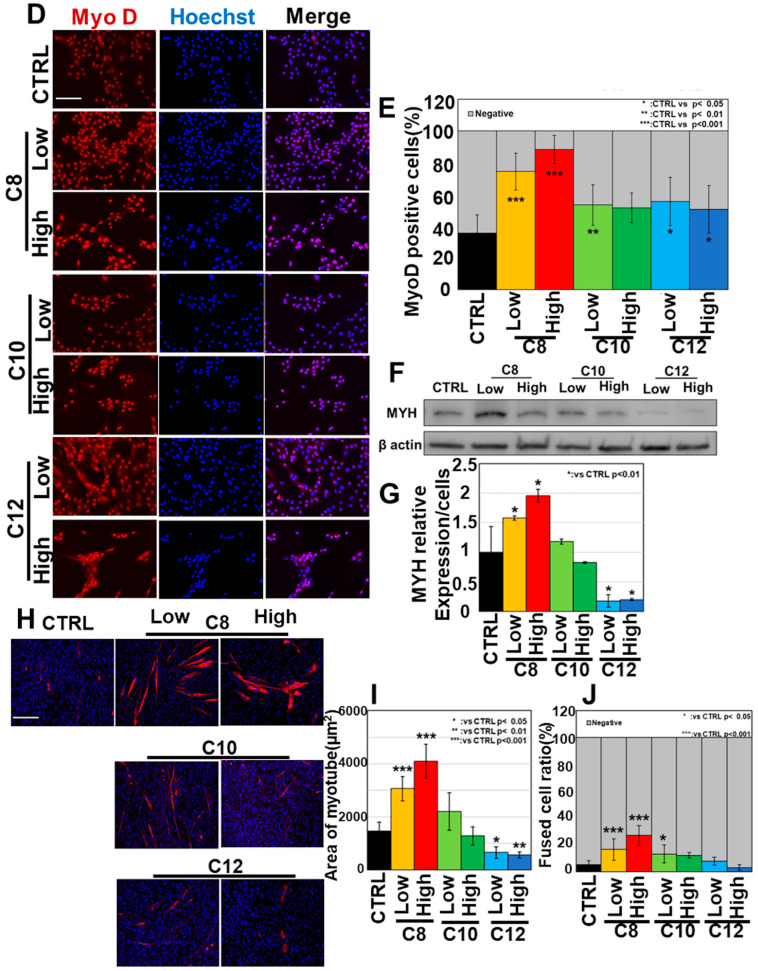
Effects of medium-chain fatty acid (MCFA) treatment on the skeletal muscle differentiation of C2C12 myotube cells. C2C12 cells were induced to myotube differentiation for 48 h with pretreatment with MCFAs (48 h). Then, the cells were examined for skeletal muscle differentiation. (**A**) Effects of MCFAs on cell proliferation. (**B**) Effects of MCFA treatments on MyoD expression levels assessed using reverse transcription–polymerase chain reaction (RT-PCR). (**C**) Semi-quantification of MyoD expression levels. (**D**) Fluorescence immunostaining of MyoD. (**E**) MyoD-positive cell rate. (**F**) MYH protein expression levels. (**G**) Semi-quantification of MYH expression levels. (**H**) MYH fluorescence immunostaining. (**I**) Semi-quantification of MYH-positive cell areas. (**J**) Fused cell ratio to all cells. Scale bar, 50 μm; error bar, standard deviation from three independent trials. Statistical differences were calculated using analysis of variance with Bonferroni correction. CTRL, control; C8, caprylic acid; C10, capric acid; C12, lauric acid; L, low; H, high; MyoD, myogenic differentiation 1; MYH, myosin heavy chain.

First, the effects of the three different MCFAs on the proliferation of skeletal myoblast C2C12 were investigated (Figure 1A). Cell proliferation was suppressed at high concentrations of all MCFAs but was promoted at low concentrations of C8 and C12. Subsequent treatments were carried out at a low concentration, where growth promotion or poor growth inhibition was observed, and at a high concentration, where growth inhibition was observed at approximately 20% inhibitory concentration (IC20). Next, the effects of MCFAs on skeletal muscle differentiation were investigated (Figure 1B,C). Examination of the effects of MCFAs on the expression of MyoD, the master gene for skeletal muscle differentiation, revealed increased expression at both low and high MCFA concentrations, especially in C8 and only at the low C10 concentration. No changes were observed for C12. The number of morphologically fused cells with multiple nuclei was most increased in C8 (high and low), slightly increased in low C10. No increase was observed in high C10 and C12 (Figure 1C lower panel). Examination of MyoD protein expression using fluorescent staining revealed high expression in C8 and low expression in C10 and C12 (Figure 1D,E). Examination of MYH expression, which is specific for myotube maturation, showed increased expression only for C8 and decreased expression for C12 (Figure 1F–I). Thus, C8 promoted skeletal muscle differentiation in MCFAs.

### 3.2. Effects of MCFAs on Mitochondrial Quality Control in C2C12 Myotubes

**Figure 2:**
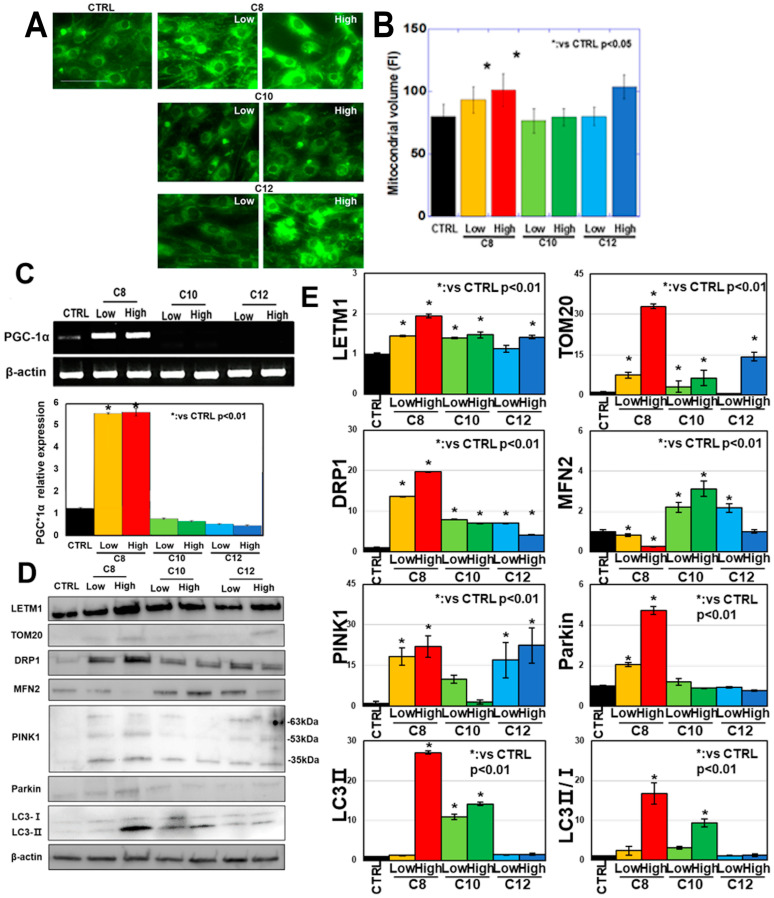

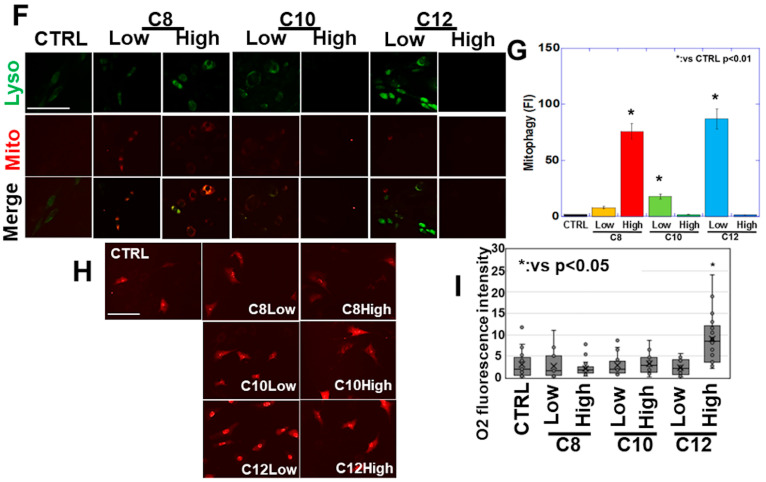
Effects of different medium-chain fatty acids (MCFAs) on mitochondrial quality control in C2C12 myotubes. C2C12 cells were induced to myotube differentiation for 48 h with pretreatment with MCFAs (48 h). Then, mitochondrial quality control was examined. (**A**) Mitochondrial volume. (**B**) Quantification of the mitochondrial volume. (**C**) PGC1α mRNA expression. The lower panel is the semi-quantification. (**D**) Mitochondrial protein expression. (**E**) Semi-quantification of panel (**C**). (**F**) Fluorescence staining for mitophagy. (**G**) Semi-quantification of panel (**F**). (**H**) Mitochondrial superoxide levels. (**I**) Semi-quantitative analysis of Panel G. Scale bar, 50 μm; error bar, standard deviation from three independent trials. Statistical differences were calculated using analysis of variance (ANOVA) with Bonferroni correction. CTRL, control; C8, caprylic acid; C10, capric acid; C12, lauric acid; PGC1α, peroxisome proliferator-activated receptor gamma coactivator 1-α; LETM1, leucine zipper and EF-hand-containing transmembrane protein 1; TOM20, translocase of the outer mitochondrial membrane 20; DRP1, dynamin-related protein 1; MFN2, mitofusin 2; PINK1, PTEN-induced kinase 1; LC3, microtubule-associated protein 1A/1B-light chain 3.

The effects of MCFAs on mitochondrial quality control were investigated in C2C12 myotubes (Figure 2). First, the changes in mitochondrial volume were examined following MCFA treatment (Figure 2A,B). The mitochondrial volume increased for both C8 concentrations and the high C12 concentration. No changes were observed for either concentration of C10 or the low concentration of C12. Examination of the expression of peroxisome proliferator-activated receptor gamma coactivator 1-α (PGC1α), a mitochondrial biogenesis-inducing gene, revealed expression only in for C8 concentration (Figure 2C). Next, the expression levels of mitochondria-related proteins were examined (Figure 2D,E). The mitochondrial inner membrane marker LETM1 and mitochondrial outer membrane marker TOM20 were increased in all MCFAs, with TOM20 showing a greater increase than LETM1, especially at the high C8 concentration. Next, dynamin-related protein 1 (DRP1), which is involved in mitochondrial fission, increased with all MCFAs, but the increase was particularly high in the high C8 concentration. In contrast, the expression of mitofusin-2 (MFN2), which is involved in mitochondrial fusion, was increased for low C10 and C12 concentrations but was decreased for low and high C8 concentrations. Furthermore, examination of mitophagy-related proteins revealed increased PTEN-induced kinase 1 (PINK1), parkin, microtubule-associated protein 1A/1B-light chain 3 (LC3), and LC3-II expression and LC3 II/I ratio at high C8 concentration. In contrast, no differences were observed in these proteins for C10 and C12. Mitophagy observed using a fluorescent marker showed enhancement for high C8 concentration (Figure 2F,G). The mitochondrial superoxide levels were enhanced at the high C12 concentration (Figure 2H,I). These findings suggest that C8 enhances mitochondrial biogenesis and autophagy, which promotes mitochondrial turnover and may contribute to mitochondrial quality control. Furthermore, the inner mitochondrial membrane increased more markedly than the outer mitochondrial membrane following treatment with C8, suggesting the induction of cristae development. In contrast, the effects of C10 and C12 on the promotion of mitochondrial quality control were ambiguous. High C12 concentration may inversely suppress mitochondrial turnover and accumulate in injured mitochondria.

### 3.3. Effects of MCFAs on Mitochondrial Respiratory Function in C2C12 Myotubes

**Figure 3:**
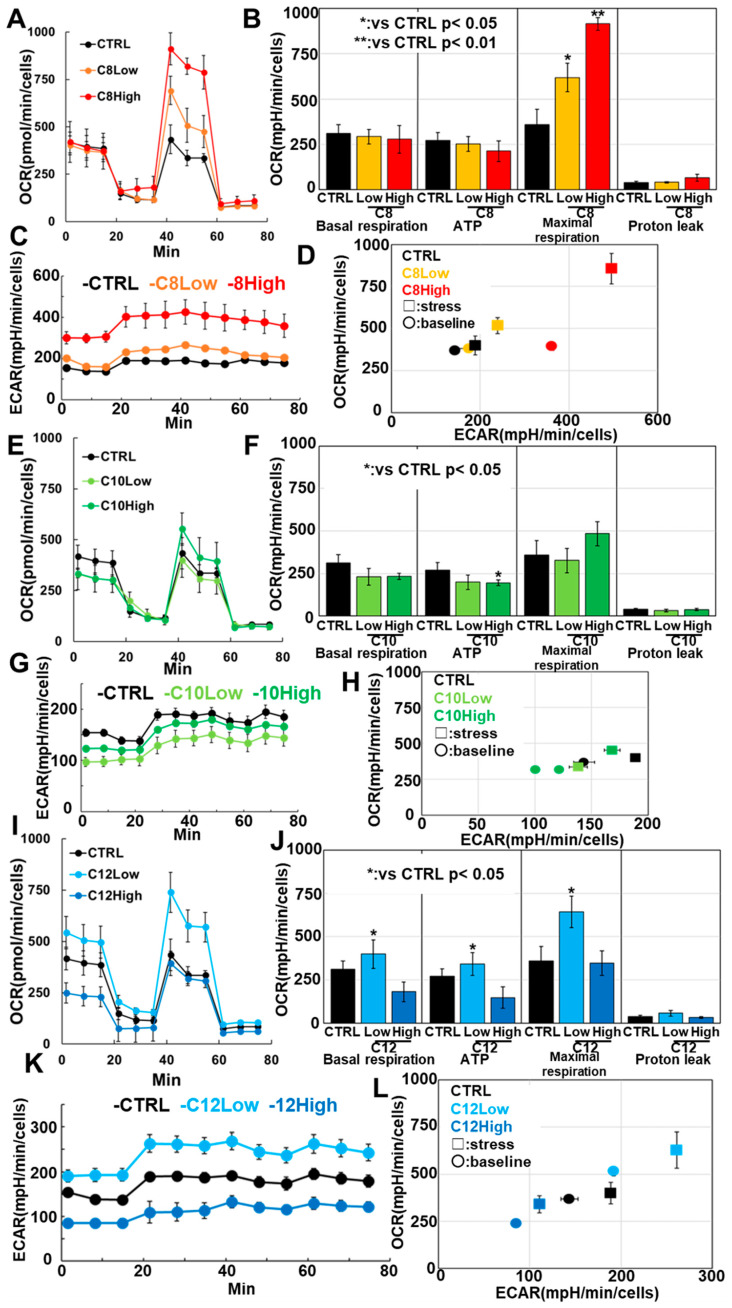
Effects of medium-chain fatty acids (MCFAs) on mitochondrial respiratory function in C2C12 myotubes. C2C12 cells were induced to myotube differentiation for 48 h with pretreatment with MCFAs (48 h). Then, the cells were subjected to respiration flux analyses. (**A**,**E**,**I**) Flux analysis. (**B**,**F**,**J**) Energy metabolic parameters calculated from the flux analysis. (**C**,**G**,**K**) ECAR analysis. (**D**,**H**,**L**) Energy metabolism phenotypes. Error bars: standard deviation from three independent trials. CTRL, control; C8, caprylic acid; C10, capric acid; C12, lauric acid; OCR, oxygen consumption rates; ECAR, extracellular acidification rate.

Next, the effects of MCFAs on the mitochondrial respiratory function of C2C12 myotubes were investigated using flux analysis (Figure 3). Neither C8 concentration caused significant changes in basal respiration, ATP production, or proton leakage, while maximum respiration increased in a concentration-dependent manner (Figure 3A,B). High C8 concentration resulted in enhanced ECAR (Figure 3C) and significant changes in energy metabolism (Figure 3D). Neither C10 concentration showed significant changes in basal respiration, maximal respiration, or proton leakage (Figure 3E,F). However, high C10 concentration resulted in reduced ATP production. C10 suppressed ECAR (Figure 3G) and led to a quiescent energy metabolic phenotype. Low C12 concentration resulted in increased basal respiration, ATP production, and maximal respiration (Figure 3I,J), as well as a weakly energetic energy metabolism phenotype. In contrast, high C12 concentration not only decreased basal respiration, ATP production, maximal respiration, and ECAR but also induced a quiescent energy metabolism phenotype (Figure 3K,L). These results suggest that C8 enhanced both mitochondrial respiratory function and glycolysis, thereby promoting energy metabolism in myotubes.

### 3.4. Effect of C8 on Enhanced Autophagy in Skeletal Muscle Differentiation

**Figure 4:**
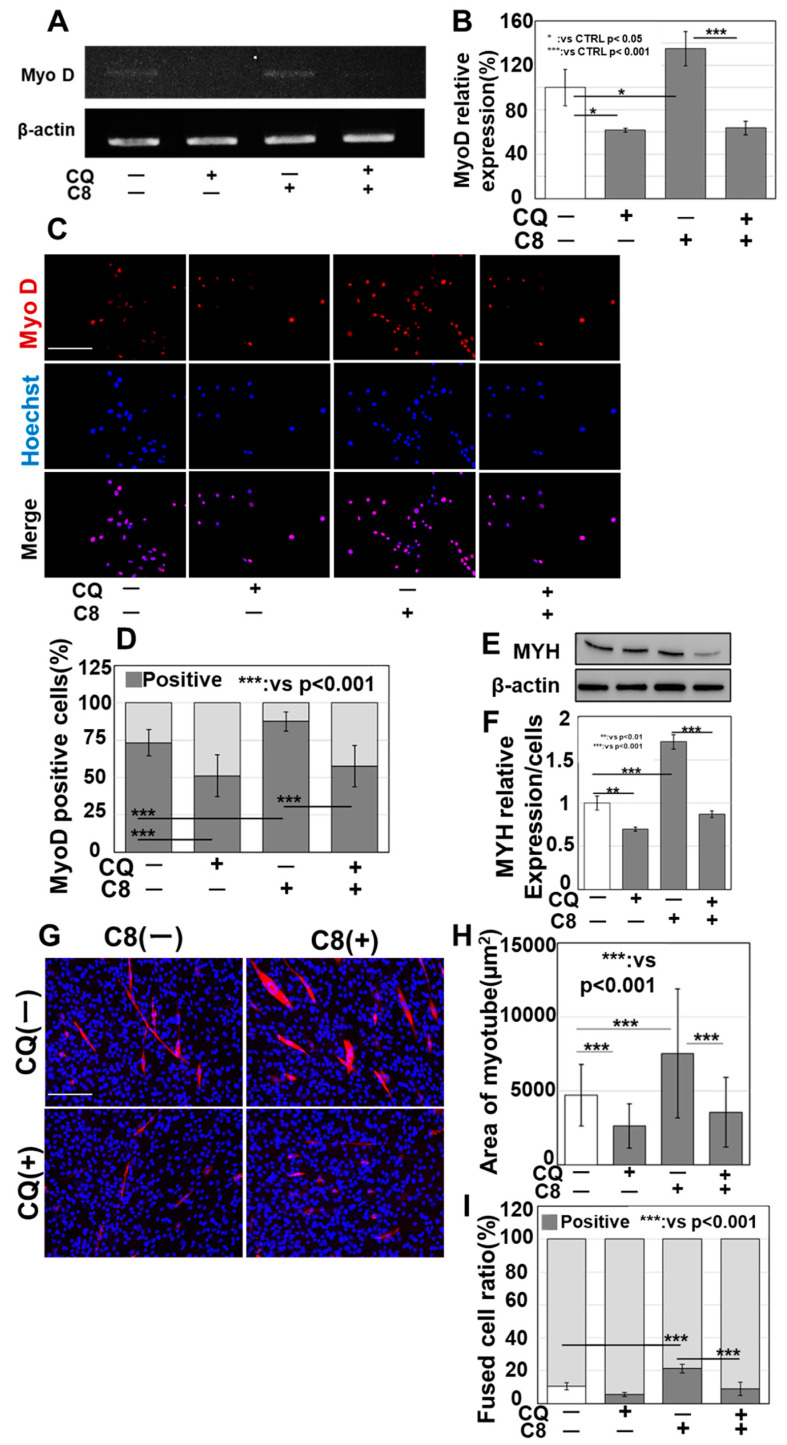
Effects of autophagy enhancement by C8 on skeletal muscle differentiation. C2C12 cells were induced to myotube differentiation for 48 h with pretreatment with C8 (48 h). We then examined the effects of C8 on skeletal muscle differentiation when autophagy was inhibited by chloroquine (CQ). (**A**) MyoD expression. (**B**) Semi-quantification of panel (**A**). (**C**) Fluorescence immunostaining for MyoD. (**D**) MyoD-positive rate. (**E**) MYH levels. (**F**) Semi-quantification of MYH protein content normalized to cell number. (**G**) MYH fluorescent fluorescence. (**H**) MYH-positive cell area. (**I**) Fused cell ratio. Scale bar, 50 μm; error bar, standard deviation from three independent trials. Statistical differences were calculated using analysis of variance (ANOVA) with Bonferroni correction. C8, caprylic acid; CQ, chloroquine; MyoD, myogenic differentiation 1; MYH, myosin heavy chain; CTRL, control; C8, caprylic acid; Hoechst, Hoechst 33342 dye.

As the above data show that C8 promoted skeletal muscle maturation and mitophagy, the effect of the C8-induced enhancement of autophagy on skeletal muscle differentiation was also investigated (Figure 4). While C8 induced MyoD expression, this effect was abrogated by the combined use of the autophagy inhibitor CQ (Figure 4A,B). MyoD protein levels showed similar results (Figure 4C,D). The C8-induced increase in MYH protein expression was abrogated by the combined use of CQ (Figure 4E–H). C8 also increased fused cells to 25% from 10% in the control (Figure 4I).

### 3.5. Impact of C8-Mediated Mitophagy on Mitochondrial Quality Control and Respiratory Function

**Figure 5:**
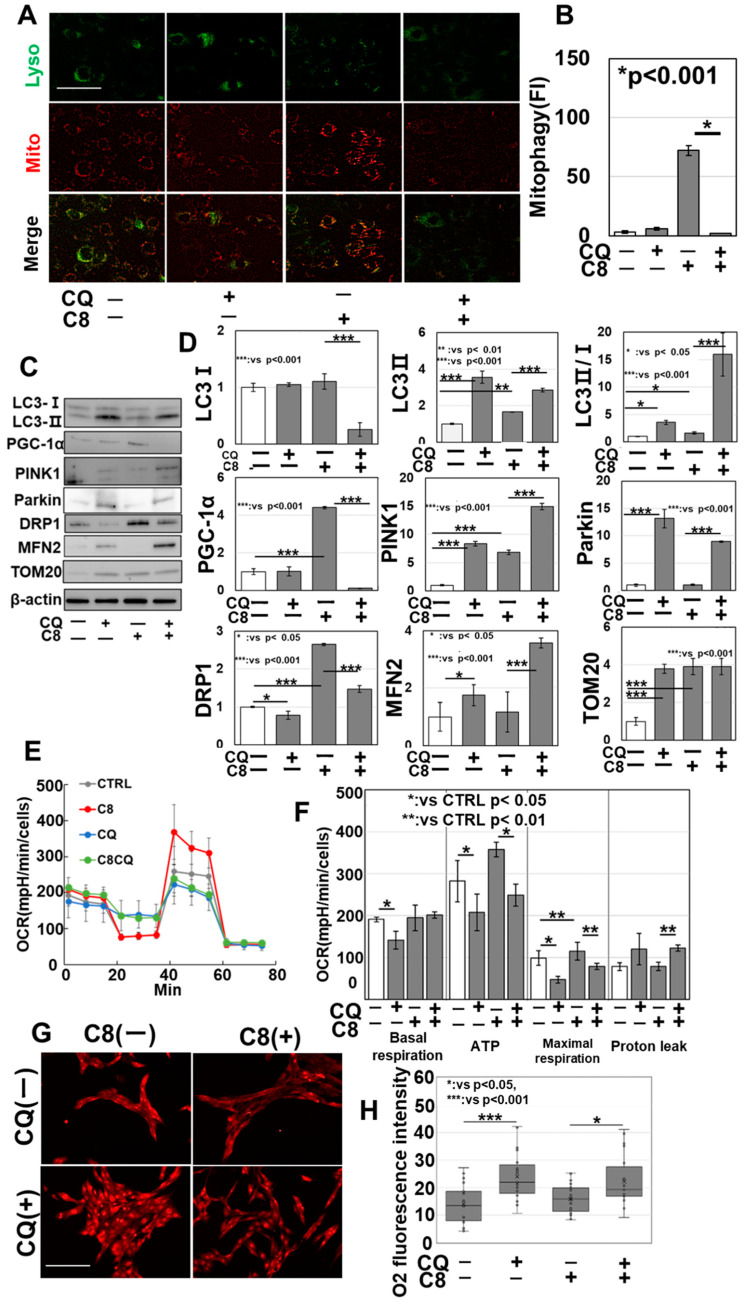
Effects of C8-induced autophagy enhancement on mitochondrial turnover. C2C12 cells were induced to myotube differentiation for 48 h with pretreatment with C8 (48 h). We then investigated the effects of C8 on myotube mitochondria when autophagy was inhibited using chloroquine (CQ). (**A**) Mitophagy fluorescence staining. (**B**) Semi-quantification of panel (**A**). (**C**) Changes in mitochondria-related protein levels. (**D**) Semi-quantification of panel (**C**). (**E**) Flux analysis. (**F**) Energy metabolic parameters calculated from the flux analysis. (**G**) Mitochondrial superoxide levels. (**H**) Semi-quantitative analysis of panel G. Scale bar, 50 μm; error bar, standard deviation from three independent trials. Statistical differences were calculated using analysis of variance (ANOVA) with Bonferroni correction. C8, caprylic acid; CQ, chloroquine; MYH, myosin heavy chain; CTRL, control; C8, caprylic acid; PGC1α, peroxisome proliferator-activated receptor gamma coactivator 1-α; TOM20, translocase of the outer mitochondrial membrane 20; DRP1, dynamin-related protein 1; MFN2, mitofusin 2; PINK1, PTEN-induced kinase 1; LC3, microtubule-associated protein 1A/1B-light chain 3.

The effects of mitophagy enhancement by C8 on mitochondrial quality control were examined (Figure 5). Mitophagy was enhanced by C8 treatment but suppressed by CQ treatment (Figure 5A,B). In addition, CQ alone increased microtubule-associated protein 2 light chain 3 (LC3II) levels, confirming the inhibition of autophagy (Figure 5C,D). Regarding proteins related to mitochondrial quality control, C8 alone increased LC3II, PINK1 (mitophagy), PGC1α (mitochondrial biogenesis), and DRP1 (mitochondrial fission) and decreased MFN2 (mitochondrial fusion) expression (Figure 5C,D). In contrast, when C8 was combined with CQ, LC3II, PINK1, and Parkin expression increased and PGC1α expression decreased compared with C8 alone. Although no significant difference was observed in TOM20, DRP1 expression was suppressed and MFN2 expression was enhanced. Evaluation of the relationship between mitophagy and mitochondrial respiratory function via flux analysis (Figure 5E,F) revealed that compared with C8 alone, the combination of C8 and CQ decreased basal respiration, maximal respiration, and ATP production and increased proton leakage. Furthermore, this combination also enhanced ROS production (Figure 5G,H). These results suggest that C8 enhanced mitophagy and promoted mitochondrial turnover and skeletal muscle differentiation.

## 4. Discussion

The most impressive finding of this study was that C8 improved mitochondrial quality control and promoted skeletal muscle cell maturation. Defective mitochondria reduce respiratory function, increase ROS accumulation, promote catabolism, and worsen muscle fiber atrophy [19]. We previously reported that mitochondrial dysfunction leads to increased ROS and decreased OXPHOS, causing myopenia in cancer cachexia [7,20]. Mitochondrial quality control consists of the contradictory processes of mitophagic removal of impaired mitochondria and mitochondrial biogenesis, both of which maintain a highly regulated balance to maintain mitochondrial number and function and cellular homeostasis, resulting in adaptation to metabolic demands and extracellular stimuli [21]. Compared with C10 and C12, C8 activated the PINK1-perkin pathway and promoted mitophagy, resulting in lower mitochondrial ROS and improved OXPHOS. Furthermore, C8 increased MyoD and MYH expression and promoted skeletal muscle maturation. The C8 diet activates the mitochondrial biogenesis pathway, increases the oxidative capacity of skeletal muscles, and induces AMP-activated protein kinase (AMPK) activation [16].

Our data show that C8 promoted mitophagy through the PINK1-parkin pathway and increased PGC1α and DRP1 expression. As a result, mitochondrial ROS levels decreased and OXPHOS was promoted. These results indicate that C8 promoted mitochondrial turnover and improved mitochondrial function. MCFAs stimulate autophagy and restore mitochondrial function, functions that are destroyed during aging and neurodegeneration [22,23,24]. Furthermore, C8 induces AMPK activation and activates the mitochondrial biogenesis pathway to enhance OXPHOS in skeletal muscle through a significant increase in PGC1a and citrate synthase gene expression and protein levels [16]. PGC1α plays a major role in the bioenergetic state of the cell, and hence protein turnover, by regulating mitochondrial biogenesis [25]. DRP1 promotes mitochondrial fission [26]. Another important function of fission is to separate and remove impaired mitochondrial components through mitophagy [27]. Thus, the activation of PGC1α and DRP1 by C8 may promote mitochondrial neogenesis and remove defective mitochondria through mitophagy, thereby promoting mitochondrial quality control. Thus, C8 may improve mitochondrial quality control and promote skeletal muscle maturation.

The present study investigated the effects of MCFAs as mitochondrial substrates. A recent study reported that MCFAs promoted mitochondrial respiratory capacity and decreased mitochondria through G-protein-coupled receptor 84 [28]. MCFAs also affect peroxisome proliferator-activated receptor gamma (PPARγ) [29,30], which has PGC1α downstream [31] and is also involved in DRP1 activation via BCL2/adenovirus E1B 19 kDa protein-interacting protein 3 [32]. Thus, PPARγ may be involved in the activation of PGC1α by C8. In addition to serving as an energy source for mitochondria, MCFAs may also be involved in actions mediated by specific receptors.

In the present study, C8 showed stronger promoting effects on mitochondrial quality control and skeletal muscle maturation compared with C12 and C10. Interestingly, differences existed among the three types of MCFAs. MCFAs are usually considered carnitine shuttle independent; however, recent studies have shown that MCFAs may depend on the carnitine shuttle, depending on the number of carbon atoms [33,34]. Among the three types of MCFAs, C8 is most easily utilized for β-oxidation. Sirtuin 5 is required for C12 oxidation but not for C10 and C8 [35]. In addition, C12 has a poorer ketogenic effect than C8 and C10 [36], while C8 has a stronger ketogenic effect than C10 [37]. In contrast, C12 exerts strong antioxidant effects via the ERK1/2/Nrf2 pathway [38]. Regarding C8 and C10, changes in the citric acid cycle, Warburg effect, glutamine/glutamate metabolism, and ketone body metabolism were observed in glioblastoma cells, and C10 showed a stronger lipogenic effect than C8 [39]. Furthermore, the anabolism of the three MCFAs into triacylglycerols is strong in C12 and almost absent in C8 [37]. These findings suggest that C8 has a stronger effect on mitochondria compared with the other MCFAs investigated in the present study.

Among the study limitations, the present study used a culture system that supplied excess glucose (4.5 g/L); therefore, linear extrapolation to living organisms may not be possible. C12 causes muscle glycogen retention by increasing fat mobilization from adipose tissue and promoting gluconeogenesis to provide post-exercise recovery from fatigue and improve aerobic endurance and muscle strength in mice [40]. In a mouse cancer cachexia model, C12 showed weak inhibition of myopenia; however, its effect was enhanced when combined with glucose loading [7]. The reason for this effect is that the β-oxidation pathway is susceptible to feedforward inhibition due to the accumulation of short-chain acyl-acetyl coenzyme A (CoA) and CoA depletion [41]. In contrast, glucose-derived acetyl-CoA regenerates free CoA and nicotinamide adenine dinucleotide (NAD+), strengthens lipid tolerance and the redox stability of cardiac mitochondria, and improves MCFA utilization [42]. This may result from the effects of the sugar combination.

In this study, muscle differentiation was induced after treatment with MCFAs, and it cannot be denied that the change in mitochondrial properties due to differentiation such as cell fusion may have altered the response to MCFAs. On the other hand, the inhibition of autophagy by chloroquine suppressed muscle differentiation, suggesting that autophagy by C8 promotes muscle differentiation via the improvement of mitochondrial quality control. This improved mitochondrial quality control may aid in the mitochondrial changes that accompany muscle differentiation. Future studies using muscle cells at various stages of muscle differentiation are needed to examine the changes in mitochondria due to muscle differentiation and the response to MCFAs.

The results of this study show that C8, unlike C10 and C12, had strong effects on promoting mitochondrial quality control and skeletal muscle differentiation and maturation. These findings suggest the potential beneficial role of C8 in the treatment and prevention of sarcopenia. However, as this research was limited to in vitro studies, future studies using animal models are needed to further investigate the clinical applications of these findings.

## Figures and Tables

**Table 1:** Primer sets, antibodies, and detection kits.

**PCR Primer**			
Gene symbol	Gene Bank ID	Forward primer (5′-3′)	Reverse primer (5′-3′)
*MyoD*	M84918.1	AGGAGCACGCACACTTCTCT	TCTCGAAGGCCTCATTCACT
*PGC-1α*	BC066868.1	AAGCACTTCGGTCATCCCTG	CAATGAATAGGGCTGCGTGC
*βactin*	BC138614.1	CTCTCAGCTGTGGTGGTGAA	AGCCATGTACGTAGCCATCC
**Antibody**			
Target	Clone or Cat#	Company	
PGC1α	#2178S	Cell Signaling Technology, Danvers, MA, USA	
LETM1	16024-1-AP	Proteintech, Tokyo, Japan	
TOM20	11802-1-AP	Proteintech, Tokyo, Japan	
PINK1	23274-1-AP	Proteintech, Tokyo, Japan	
Parkin	#4211	Cell Signaling Technology, Danvers, MA, USA	
LC3	PM036	Medical & Biological Laboratories, Tokyo, Japan	
DRP1	#8570	Cell Signaling Technology, Danvers, MA, USA	
MFN2	9482S	Cell Signaling Technology, Danvers, MA, USA	
MYH	sc-376157	Santa Cruz Biotechnologies, Santa Cruz, CA, USA	
MyoD	sc-377460	Santa Cruz Biotechnologies, Santa Cruz, CA, USA	
β-actin	sc-47778	Santa Cruz Biotechnologies, Santa Cruz, CA, USA	
**Detection Kit**			
Target	Catalog number	Company	
Mitophagy	344-91901	Dojindo, Kumamoto, Japan	
mitoGreen	346-92061	Dojindo, Kumamoto, Japan	
MitoROS	16052	Sigma Life Science, St. Louis, MO, USA	

PGC1α, peroxisome proliferator-activated receptor gamma coactivator 1-α; MyoD, myogenic differentiation 1; LETM1, leucine zipper and EF-hand-containing transmembrane protein 1; TOM20, translocase of the outer mitochondrial membrane 20; DRP1, dynamin-related protein 1; MFN2, mitofusin 2; PINK1, PTEN-induced kinase 1; LC3, microtubule-associated protein 1A/1B-light chain 3.

## Data Availability

Data are contained within the article.

## Abbreviation

MCFA: medium-chain fatty acid; ROS, reactive oxygen species; OXPHOS, oxidative phosphorylation; LCFA, long-chain fatty acid; C12, lauric acid; C10, capric acid; C8, caprylic acid; OCR, oxygen consumption rates; ECAR, extracellular acidification rate; TCA, tricarboxylic acid; MYH, myosin heavy chain; MyoD, myogenic differentiation 1; CQ, chloroquine; PGC1α, peroxisome proliferator-activated receptor gamma coactivator 1-α; DRP1, dynamin-related protein 1; MFN2, mitofusin-2; PINK1, PTEN-induced kinase 1; LC3, microtubule-associated protein 1A/1B-light chain 3; LC3II, microtubule-associated protein 2 light chain 3; AMPK, AMP-activated protein kinase; PPARγ, proliferator-activated receptor gamma; NAD+, nicotinamide adenine dinucleotide.
